# The impact of the SARS‐CoV‐2 pandemic on global influenza surveillance: Insights from 18 National Influenza Centers based on a survey conducted between November 2021 and March 2022

**DOI:** 10.1111/irv.13140

**Published:** 2023-05-11

**Authors:** Lisa Staadegaard, Marco Del Riccio, Sytske Wiegersma, Clotilde El Guerche‐Séblain, Erica Dueger, Meral Akçay, Jean‐Sebastien Casalegno, Michel Dückers, Saverio Caini, John Paget, The NIC Collaborators, Vina Lea Arguelles, Inmaculada Cases, Julian Druce, Dominic E Dwyer, Joanna Ellis, José María Eiros, Ron Fouchier, Le Thi Quynh Mai, Michal Mandelboim, Adam Meijer, Pilailuk Okada, Varsha Potdar, Simona Puzelli, Marilda Siqueira, Cao Minh Thang, Martine Valette, David E. Wentworth, Nicole Wolter

**Affiliations:** ^1^ Netherlands Institute for Health Services Research (Nivel) Utrecht The Netherlands; ^2^ Postgraduate Medical School in Public Health University of Florence Florence Italy; ^3^ Sanofi, Global Medical Influenza Franchise Lyon France; ^4^ Hospices Civils de Lyon, Hôpital de la Croix‐Rousse, Centre de Biologie Nord, Institut des Agents Infectieux, Laboratoire de Virologie Lyon France; ^5^ ARQ National Psychotrauma Centre Diemen The Netherlands; ^6^ Faculty of Behavioural and Social Sciences University of Groningen Groningen The Netherlands

**Keywords:** epidemics, influenza, public health, SARS‐CoV‐2, surveillance

## Abstract

**Background:**

National Influenza Centers (NICs) have played a crucial role in the surveillance of SARS‐CoV‐2. The FluCov project, covering 22 countries, was initiated to monitor the impact of the SARS‐CoV‐2 pandemic on influenza activity.

**Methods:**

This project consisted of an epidemiological bulletin and NIC survey. The survey, designed to assess the impact of the pandemic on the influenza surveillance system, was shared with 36 NICs located across 22 countries. NICs were invited to reply between November 2021 and March 2022.

**Results:**

We received 18 responses from NICs in 14 countries. Most NICs (76%) indicated that the number of samples tested for influenza decreased. Yet, many NICs (60%) were able to increase their laboratory testing capacity and the “robustness” (e.g., number of sentinel sites) (59%) of their surveillance systems. In addition, sample sources (e.g., hospital or outpatient setting) shifted. All NICs reported a higher burden of work following the onset of the pandemic, with some NICs hiring additional staff or partial outsourcing to other institutes or departments. Many NICs anticipate the future integration of SARS‐CoV‐2 surveillance into the existing respiratory surveillance system.

**Discussion:**

The survey shows the profound impact of SARS‐CoV‐2 on national influenza surveillance in the first 27 months of the pandemic. Surveillance activities were temporarily disrupted, whilst priority was given to SARS‐CoV‐2. However, most NICs have shown rapid adaptive capacity underlining the importance of strong national influenza surveillance systems. These developments have the potential to benefit global respiratory surveillance in the years to come; however, questions about sustainability remain.

## 1 INTRODUCTION

The emergence of a new respiratory infection (COVID‐19) in early 2020 has had a profound impact on the activity and surveillance of other respiratory infections including influenza.^1^ Influenza causes seasonal epidemics in most parts of the world and is responsible for a high burden of disease.^2^, ^3^, ^4^ In temperate climates, influenza activity generally peaks in colder, winter months. However, during the Northern Hemisphere's winter of 2019/2020, seasonal influenza activity was dramatically interrupted with limited to no activity reported in most countries in both hemispheres.^1^, ^5^, ^6^ In contrast, during the Southern Hemisphere winter of 2021 and Northern Hemisphere winter of 2021/2022, many countries saw co‐circulation of both SARS‐CoV‐2 and influenza. However, the levels of influenza activity during winter remained far lower compared with pre‐COVID years.^6^ Yet, several countries (e.g., France and South Africa) experienced a second wave of influenza activity outside of the typical influenza season.^7^ High levels of influenza activity returned for several Southern Hemisphere countries in 2022 with Australia experiencing higher weekly case counts compared with the 5‐year average.^8^, ^9^ A variety of factors are thought to have contributed to these unusual patterns of influenza activity. These include the introduction and/or relaxation of non‐pharmaceutical interventions (NPIs), such as travel regulations, mask wearing, viral interference as well as high (er) influenza vaccination rates, and changes in care‐seeking behavior or patient pathways.^10^, ^11^, ^12^, ^13^, ^14^

The “Global Influenza Surveillance and Response System” (GISRS), managed by the World Health Organization (WHO), is the primary global mechanism and resource for the surveillance and control of influenza.^15^, ^16^ This system was established in 1952, celebrating its 70th birthday in 2022, and currently encompasses 146 National Influenza Centers (NICs) based in 123 countries.^17^, ^18^ NICs play a critical role in pandemic influenza risk assessment and the WHO recommendations for the influenza vaccine composition by conducting and sharing surveillance data to the FluNet platform.^16^, ^19^, ^20^ FluNet is a dataset that provides publicly available, national level, weekly influenza surveillance data. All NICs adhere to common protocols/regulations and are periodically evaluated. Yet, major differences in the characteristics of their surveillance system exist (e.g., geographical representativeness or case definitions). These differences can impact the quality and representativeness of data reported to FluNet.^21^ In addition, the reliability, completeness, and accuracy of the influenza surveillance data are largely driven by national healthcare infrastructures and appear positively associated with a higher number of NICs as well as participating (sentinel) sites and greater health expenditures.^22^

Since the beginning of the pandemic, these national influenza surveillance systems have been leveraged for the surveillance of the SARS‐CoV‐2 virus.^20^ As a result, many NICs became COVID‐19 testing centers that functioned alongside national public, and private sector COVID‐19‐specific testing centers.^23^ To monitor the impact of the SARS‐CoV‐2 pandemic on influenza activity and surveillance, the FluCoV project (nivel.nl/en/flucov) was initiated in 2021. As part of this project, we published a monthly epidemiological bulletin portraying SARS‐CoV‐2 activity alongside influenza in 22 countries around the world. In addition, the project consisted of a survey to be circulated digitally among NICs located in the countries portrayed in the bulletin. The survey aimed to increase our understanding of national influenza surveillance structures and how these were affected by the COVID‐19 pandemic. Here, we present the influenza surveillance data we retrieved as part of the FluCov project in combination with the survey outcomes and discuss the overall impact the pandemic has had on influenza surveillance including the quality of surveillance data and the likely future of influenza surveillance.

## 2 METHODS

The FluCov project covers 22 countries distributed over the American (Canada, Brazil, Mexico, and the USA), African (South Africa), European (France, Germany, Italy, Israel, Netherlands, Poland, Spain, and the United Kingdom), Eastern Mediterranean (Egypt), South East Asian (India and Thailand), and Western Pacific regions (Philippines, Australia, China, Vietnam, Japan, and South Korea). Countries were chosen based on their geographical spread, the presence of a NIC, and the consistent reporting of data to FluNet. In the participating countries, 36 individual NICs were located. As part of this project, we conducted a survey and publish a monthly epidemiological bulletin. These bulletins have been published since June 2021 and provide an overview of the number of positive cases of influenza and SARS‐CoV‐2 and the percentage of specimens testing positive from January 2019 onwards.^6^ The current paper focuses only on the influenza surveillance data extracted as part of this project in combination with the NIC survey.

### 2.1 Surveillance data extraction

Influenza data on weekly case numbers and number of specimens taken were extracted from FluNet.^24^ The data are provided by NICs and other national influenza reference laboratories collaborating actively with GISRS or are uploaded from WHO regional databases. Data were extracted from the FluNet platform at the beginning of each month since June 2021, and historical data were extracted from the 1st of January 2019 onwards. Data from this platform are regularly updated and sometimes retrospectively corrected.

### 2.2 National Influenza Centers survey

To better understand (evolution of) the surveillance data reported by the NICs during the pandemic, we designed a survey to be filled out by a NIC representative (see Supporting Information) using surveyplanet.com.^25^ The survey consisted of two parts to be filled out (a) by those who report influenza surveillance data to the FluNet platform or (b) by those who report SARS‐CoV‐2 surveillance data to the WHO. A draft version of the survey was reviewed by two NIC collaborators as well as shared with the Global Influenza Program team at the WHO for feedback after which we incorporated their suggestions.

NICs were invited to reply to the survey between November 2021 and March 2022. Contact details for NIC contact persons were retrieved by internet searches. If a provided answer was unclear, we retrospectively contacted the participating NIC for clarification. Survey results were agreed to be treated confidentially and are thus reported as aggregated responses (i.e., data are not presented by country).

## 3 RESULTS

### 3.1 Influenza activity

**FIGURE 1:**
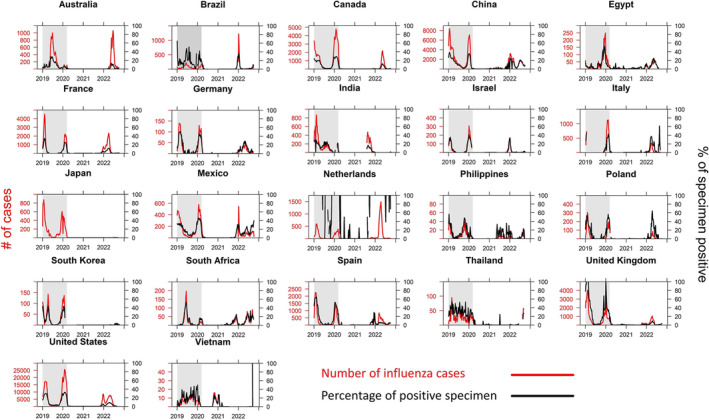
Influenza circulation as reported to FluNet for 22 countries (2019–2022). Grey shaded area indicates the time before COVID‐19 was characterized as a pandemic (11/03/2020)^26^

Between June 2021 and September 2022, 14 FluCoV epidemiological bulletins were produced and published online. Overall, the data showed limited influenza activity since the start of the SARS‐CoV‐2 pandemic (Figure 1). For most temperate Northern Hemisphere countries, the 2019/2020 influenza epidemic was just coming to an end once cases of SARS‐CoV‐2 started to rise in March 2020. In these regions, little to no new influenza cases occurred during the typical influenza season (between late December and February) for the 2020/2021 season.^27^ The only exception was China, where the first rise of SARS‐CoV‐2 cases coincided with their influenza epidemic. Temperate countries in the Southern Hemisphere that typically experience influenza activity between April and September^27^ experienced very limited or no influenza activity for both the 2020 and 2021 seasons. The end of 2021 was the first instance in which many countries in both hemispheres experienced substantial influenza activity, and during the course of 2022, we saw many temperate countries experiencing atypical peaks in influenza activity in summer, outside of the regular season. Generally, influenza case numbers reported to FluNet remained lower than expected with a few exceptions showing higher case counts for the 2021/2022 or 2022 winter (e.g., Australia, Brazil, and The Netherlands).

### 3.2 Survey results

**FIGURE 2:**
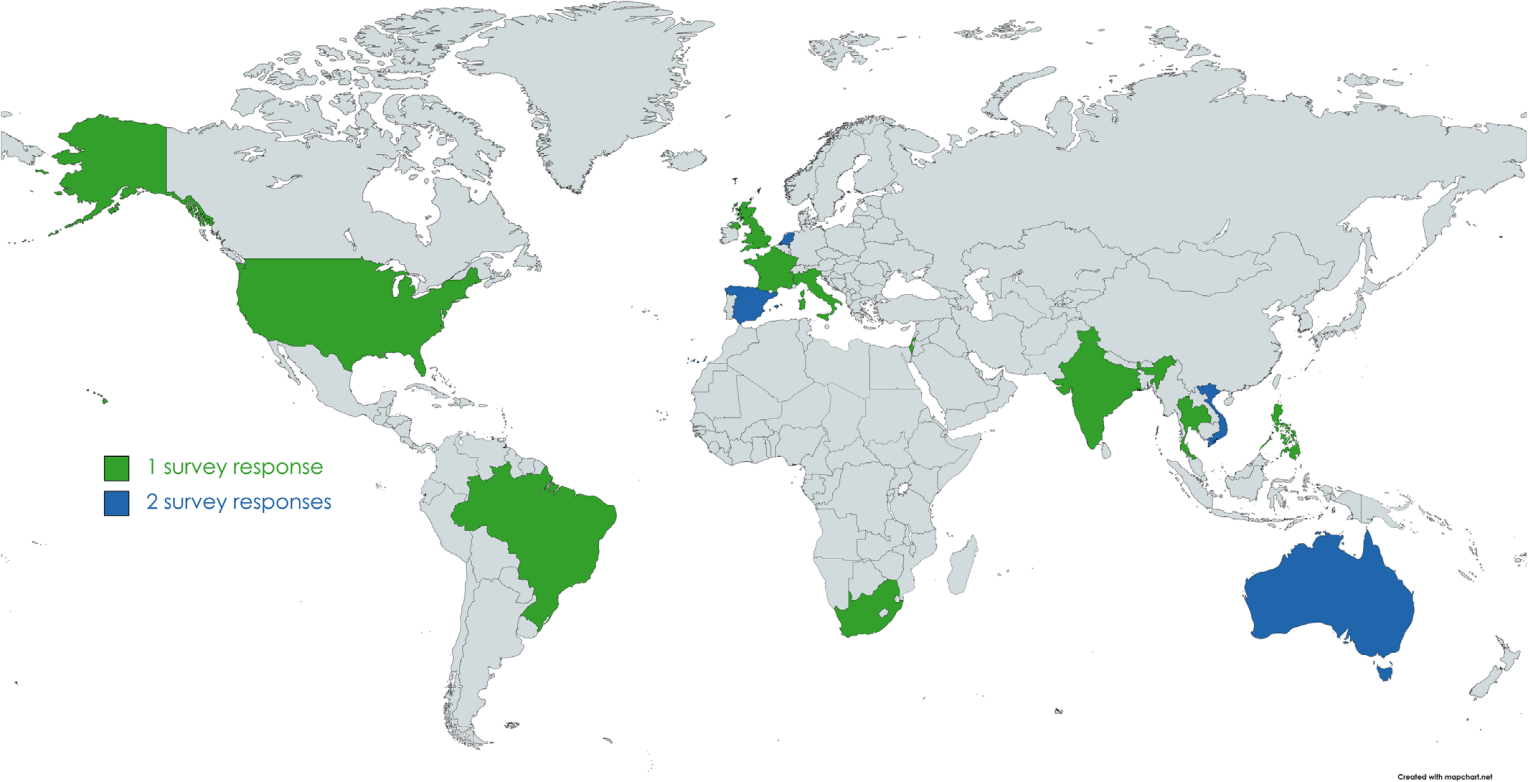
Map portraying the origin of the NICs that replied to the survey. For countries in green, we had one participating NIC, and countries in blue had two. Below figure: Countries listed are Australia, Brazil, France, India, Israel, Italy, Netherlands, Philippines, South Africa, Spain, Thailand, United Kingdom, United States, and Vietnam.

Between November 2021 and March 2022, we retrieved contact details and approached 30 different NICs in the participating countries to complete the NIC survey. Of these, we received 18 responses (response rate: 60%) from NICs in 14 countries. For the Netherlands, we received two responses from the two institutes in charge of influenza surveillance. Officially, their combined efforts form one NIC—but in this paper, we have considered their responses separately. Participating countries are shown in Figure 2, and their characteristics can be found in Table 1. Five of these countries are low‐ or middle‐income countries (LMICs), and the other nine countries are all high‐income countries (HICs) according to the World Bank income classification.^28^

**TABLE 1:** Basic characteristics of participating National Influenza Centers (*n* = 18).

Characteristics	Number (%)
Hemisphere	
Northern	14 (78%)
Southern	4 (22%)
WHO region	
African Region	1 (6%)
Region of the Americas	2 (11%)
South‐East Asian Region	2 (11%)
European Region	8 (44%)
Eastern Mediterranean Region	0 (0%)
Western Pacific Region	5 (28%)
World Bank Income category	
High	12 (67%)
Middle or low	6 (33%)
Reports influenza data to FluNet	
Yes	17 (94%)
No	1 (6%)
Reports SARS‐CoV‐2 data to WHO	
Yes	10 (56%)
No	8 (44%)

Of these 18 responses, 17 indicated to report their influenza data to the FluNet database and as such completed the influenza‐related section of the questionnaire.

#### 3.2.1 Influenza sample sources and representativeness of population

**FIGURE 3:**
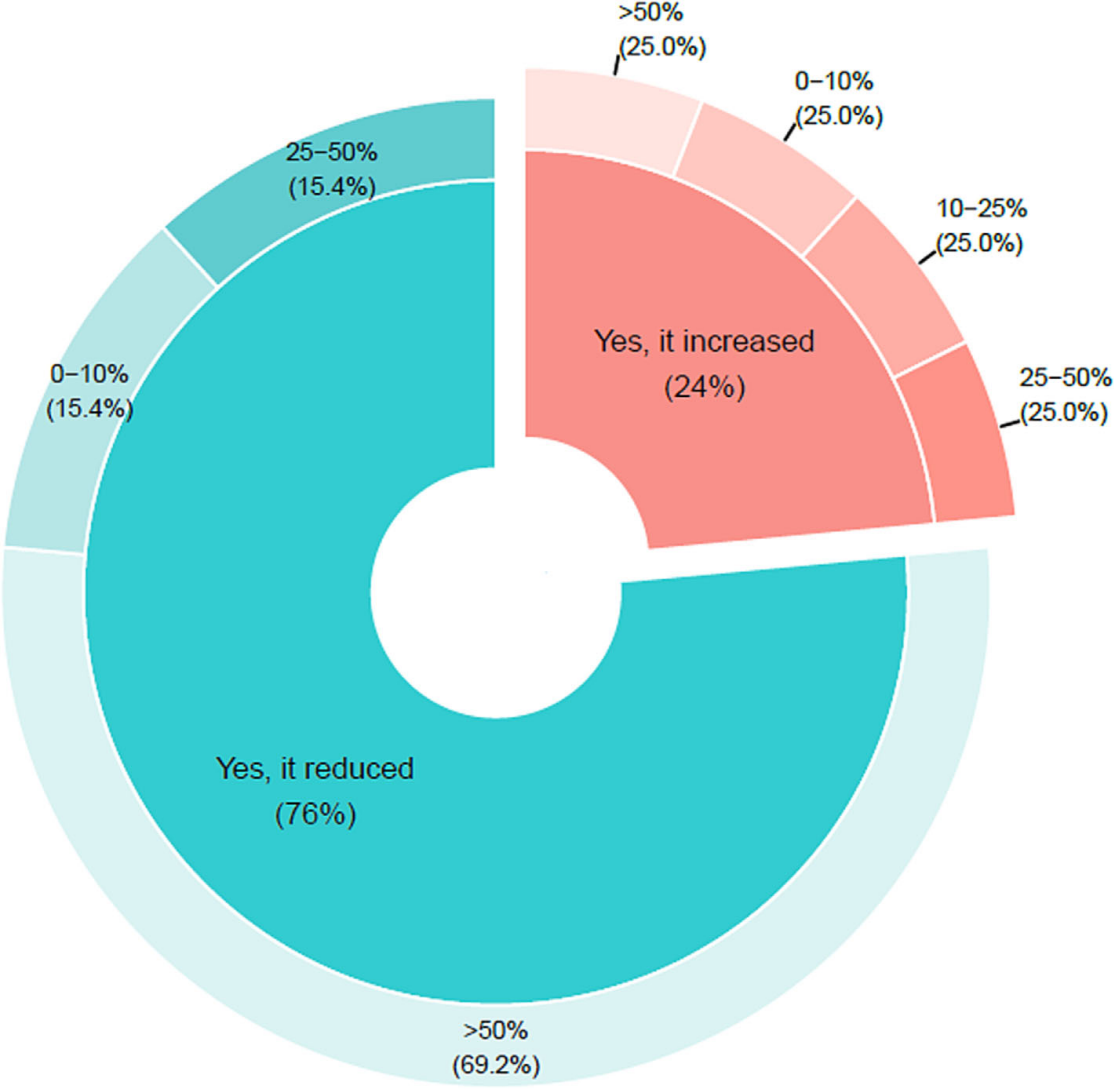
Responses to the Survey Question: Has there been a change in the total number of specimens tested for influenza since the SARS‐CoV‐2 pandemic? (*n* = 17).

Most responses (76%, 13/17) indicated that the number of samples being tested for influenza since the start of the pandemic was reduced, and nine of those reported a reduction of ≥50% (Figure 3). Of the countries reporting an increase in samples tested for influenza (24%, 4/17), one reported an increase of ≥50%, one between 25% and 50%, one between 10% and 25%, and one reported an increase of 0%–10%. Responses from NICs in LMIC did not differ with 66% (4/6) reporting a decrease in the number of samples tested for influenza, of which three reported that this was a reduction of ≥50%.

Even before the COVID‐19 pandemic, the sources (e.g., hospitalized care or general practitioners [GP]) of samples to be tested for influenza uploaded onto FluNet per country varied widely (Table S1). Following the COVID‐19 pandemic onset, most NICs (*n* = 14) saw a shift in sample origin, with only two NICs (see responses 8 and 14; Table S1) reporting no shifts in surveillance sample sources. No distinct pattern in these shifts could be identified. Whereas some NICs report that the majority source of samples shifted toward the hospital setting, others reported that the majority of samples were now taken from the outpatient setting or a different distribution all together.

The majority (59%, 10/17) of NICs reported that their influenza system has been “structurally” changed since the start of the pandemic; this proportion was similar in HICs (55%, 6/11) and LMICs (67%, 4/6). Six NICs reported no “structural” changes have taken place since the onset of the pandemic, and one NIC replied not knowing if “structural” changes occurred. Those reporting changes gave several reasons for this including increased number of sentinel sites (*n* = 2), different distribution of sentinel sites (e.g. GP v. hospital, *n* = 3), and changes in geographical representativeness (*n* = 2). For one NIC, this meant that the GP surveillance sites increased from 40 to 140 practices, well distributed across the country. One NIC also reported an increase in the use of point‐of‐care testing in hospitals as well as increased self‐sampling and an increased number of studies taking place as part of the surveillance system.

#### 3.2.2 Laboratory practices (capacity and diagnostics)

Most NICs (59%, 10/17) indicated that their surveillance system had become more “robust” since the start of the pandemic, as was the case for five of the six NICs in LMICs. Overall, five NICs (29%, 5/17) indicated that the robustness of their surveillance system had not increased, and two NICs indicated that they did not know if any changes occurred since the start of the pandemic. Of NICs reporting an increase in robustness, most (70%, 7/10) reported that this was in part due to increased funding available for personnel and equipment since the pandemic and some NICs reported a higher number of sentinel sites (30%, 3/10). Thirteen out of 17 NICs (76%) reported that good laboratory practices (e.g., quality control) had not been impacted following the pandemic. Of the participating NICs, 94% (16/17) reported testing (some of) the influenza samples for SARS‐CoV‐2. Nine of these NICs reported doing so for all the influenza samples, whereas the other seven only tested a subset of samples for SARS‐CoV‐2. Only one of the included NICs did not test influenza samples for SARS‐CoV‐2. All NICs based in LMICs indicated testing (some) of their influenza samples for SARS‐CoV‐2.

The laboratory influenza testing capacity of NICs varied widely even prior to the pandemic, with capacity ranging from two samples to 1000 samples per week (Table 2). Overall, capacity was highest in NICs located in HICs compared with LMICs, but both were able to scale up their capacity following the pandemic.

**TABLE 2:** Changes in the influenza weekly/monthly testing capacity per NIC as a consequence of the pandemic.

Income level	#	Influenza testing capacity prior to the pandemic	Influenza testing capacity during the pandemic
High	**1**	100/month	100/month
	**2**	1000/week	5000/week
	**3**	NA	NA
	**4**	Maximum 500/week at peak of epidemic season	Maximum 1000/week at peak of epidemic season
	**5**		no difference.
	**6**	650/week routinely, but up scalable	2500/week routinely, but up scalable
	**7**	600/week	1500/week
	**8**	500/week routinely, but up scalable	500/week routinely, but up scalable
	**9**	200/week	200/week
	**10**	1000/week	2500/week
Middle or low	**11**	200/week	200/week
	**12**	100–150/week	Maximum 400/week
	**13**	200/week	200/week
	**14**	50–100^a^	300–500^a^
	**15**	500/week	3000–5000/week
	**16**	200/week	500/week

^a^ Units (e.g., week and month) were missing.

For several NICs (60%, 9/15), this testing capacity increased following the start of the pandemic; for six others, the capacity remained the same. In LMICs, the testing capacity mostly (67%, 4/6) increased, with two NICs in LMICs reporting no difference in influenza testing capacity since the start of the pandemic. For NICs that reported a change in diagnostics methodology over the course of the pandemic (29%, 5/17), all reported that this change was due to the use of multiplex real‐time polymerase chain reaction (RT‐PCR) assays that detect both SARS‐CoV‐2 and influenza. The majority of respondents (76%, 13/17) reported seeing increased capacity to sequence the influenza‐positive samples using Next Generation Sequencing (NGS) and all but one NIC (94%, 15/16) reported uploading their sequencing data to the Global Initiative on Sharing All Influenza Data (GISAID).

#### 3.2.3 Burden of work

All NICs (16/16) indicated they had seen an increase in the burden of work as a consequence of the pandemic. Several NICs (19%, 3/16) reported that this resulted in the hiring of additional staff or by outsourcing some of the work to other institutes, departments, or staff not usually involved in these activities.

Ten of 17 NICs reported that their collaboration with the WHO had changed since the start of the pandemic, whereas five reported the pandemic had had no impact on this collaboration. Those saying the collaboration was altered mainly indicated an increased number of meetings and guidelines with all of these occurring virtually. One of the NICs stated that “the number of meetings exploded,” and another noted that “Sometimes duplication by different WHO groups. Hard to keep up with all the different groups.”

#### 3.2.4 Future of laboratory surveillance for influenza

We asked participants how they envision influenza surveillance will evolve or change in the coming 2–5 years in their respective countries. Many (47%, 8/17) of the responses included the possibility of integrating SARS‐CoV‐2 surveillance into the previously existing respiratory surveillance system. Other answers included the transfer of surveillance to the hospital, restoring of sentinel GP networks, increased use of syndromic testing as well as point‐of‐care testing, less culture and more PCR testing, increased sequencing capacity, and one NIC indicated they do not foresee major changes in the near future. One of the NICs indicated that the sustainability of the increased number of laboratories with sequencing capacity will be a challenge for the future. Another NIC indicated that the genomic surveillance of SARS‐CoV‐2 has enabled them to conduct faster genome sequencing and improved bioinformatics analysis for public health action, processes that they plan to apply to the analysis of influenza surveillance data as well.

#### 3.2.5 Surveillance for SARS‐CoV‐2

Ten of the NICs participating in the survey shared their SARS‐CoV‐2 surveillance data with the WHO, four of which are located in LMICs. These NICs were invited to fill out the second part of our survey. The majority of the NICs started reporting SARS‐CoV‐2 data as of early 2020, with only one NIC fulfilling this role as of January 2021. Most NICs (70%, 7/10) reported both diagnostic and sequencing data and all of them uploaded their sequencing data to the SARS‐CoV‐2 section of GISAID. The sources of the samples that the NICs tested for SARS‐CoV‐2 can be found in Table S2.

Five out of 11 NICs reported that the population from which specimens are tested for SARS‐CoV‐2 had changed. Reported reasons for this change included “a shift from GP surveillance to municipal health service testing streets,” “decrease in the number of samples received from GPs,” “switch from hospital to community outbreaks,” and “universal surveillance is implemented.” Four out of 10 NICs reported that they had been forced to reduce resources allocated to influenza surveillance activities in order to meet the demand for SARS‐CoV‐2 activities, with two of these NICs based in LMICs. Five NICs also reported changes in reporting practices (e.g., proportion of sentinel vs. non‐sentinel samples received) for SARS‐CoV‐2, with two of these mentioning the implementation of the Acute Respiratory Infection (ARI) case definition. One NIC reported a decrease in the amount of metadata coming from the new reporting structures, and one reported not having time to review the data.

## 4 DISCUSSION

The SARS‐CoV‐2 pandemic has had a profound impact on influenza activity as well as the national and global influenza surveillance system.^6^, ^29^, ^30^ In line with other literature, the FluCov epidemiological bulletins showed how the timing and intensity of influenza activity were atypical and often limited between 2020 and 2022.^6^ Yet our survey results also indicate that many NICs saw a substantial reduction in the number of specimens being tested for influenza since the start of the pandemic. Although this decrease likely also resulted from ongoing low rates of influenza circulation,^29^ it suggests that for many NICs, the surveillance of influenza was (temporarily) halted or disrupted, whilst priority was given to the SARS‐CoV‐2 pandemic.

### 4.1 Impact of SARS‐CoV‐2 pandemic on national surveillance data

The NICs play a crucial role in the surveillance of influenza at a national level, and this involves many different activities such as the testing of samples, performing strain characterizations, providing specimens to the WHO reference laboratories for the vaccine strain selection procedure, writing national surveillance bulletins, and carrying out or supporting research projects.^17^

Our survey shows how these national systems differed greatly across countries prior to the SARS‐CoV‐2 pandemic and that surveillance activities were variously disrupted as a consequence of the pandemic response efforts. We found that the sources of samples, the geographic and demographic representatives, and the diagnostic methods differed. All of these factors were also impacted by the pandemic, with the sources of samples shifting (e.g., hospitalized or community care), changes in the representativeness of these samples, and an increased availability of PCR testing. These findings are confirmed by a recent study showing that minimal to major adaptations were made to sentinel surveillance systems as a response to the pandemic in seven European countries.^14^ This study showed that changes varied per country, with some discontinuing all respiratory surveillance activities and others altering their patient pathways (e.g., parallel testing routes) or sampling criteria.^14^ Although our results show that most NICs were able to respond to the pandemic quickly by scaling up and reallocating resources, this often (temporarily) came at the cost of testing for influenza and was accompanied by an increase in the burden of work across all included NICs. However, overall our results indicate that the consequences of these efforts have the potential to benefit the NICs in the long term (e.g., in terms of capacity building). A similar effect was seen after the influenza A/H1N1 2009 pandemic when reporting completeness improved significantly.^22^, ^31^

### 4.2 Impact of the SARS‐CoV‐2 pandemic on the global WHO FluNet surveillance data

The COVID‐19 pandemic has impacted the global influenza surveillance data as presented through FluNet in several ways. Our monthly FluCoV bulletins have highlighted how most NICs reported limited to no influenza cases during the typical epidemic period(s) in both the Northern (2020/2021 season) and Southern Hemisphere (2020 and 2021 seasons) following the pandemic, with the 2021/2022 season being the first in which many countries in both hemispheres experienced substantial influenza activity.^32^ As confirmed through our questionnaire, overall, the pandemic (temporally) impacted the ability of NICs to perform continuous and reliable influenza testing, with lower numbers of samples being processed.^1^, ^23^ The scale of this decrease was large, with the majority of NICs reporting a ≥50% decrease. Yet, plenty of evidence exists confirming that influenza circulation was indeed limited as a consequence of the pandemic, as shown in studies comparing several surveillance systems and countries in which sampling for influenza actually increased.^33^, ^34^, ^35^

It is worth considering how reliable the influenza surveillance data from January 2020 to March 2022 were in this context and what implications our findings have for the interpretation of current surveillance data. Not only were NICs unable to provide continued surveillance efforts as a consequence of a higher work burden and redistribution (or lack) of resources (e.g., personnel and materials), NPIs in the form of movement restrictions or stay‐at‐home orders have also affected people's behavior or their ability to access care.^1^ This means that the lower influenza case counts, albeit largely caused by limited influenza circulation, could have also been caused by changes in the structure of the surveillance system^33^ or decreases in syndromic consultations.^1^ These factors should be considered when interpreting influenza surveillance data that were collected during the SARS‐CoV‐2 pandemic and may affect the reliability of the data presented through FluNet.

### 4.3 Strengthening the influenza surveillance systems since the emergence of SARS‐CoV‐2

Though the influenza surveillance systems appeared to have been disrupted in the majority of NICs, in most instances, the structural changes that are reported have the potential to benefit influenza surveillance in the long term.^36^ The majority of NICs reported an increase in the capacity of samples that they can now test for influenza and 70% reported an increase in “robustness.” This robustness takes several forms among which are increased geographical representatives, increased number of sentinel sites, and increased availability of funding for equipment and personnel. In addition, we found an increased availability of PCR testing, the importance of which was underlined by the pandemic.

The roll‐out of additional reporting systems, increases in sentinel sites, and better geographic representativeness as a result of the SARS‐CoV‐2 pandemic have the potential to help provide a better picture of the epidemiology and overall burden of influenza at a national level in the future.^36^ The increased availability of PCR testing, including multiplex testing, will also aid this and could assist in creating improved recommendations for influenza and likely SARS‐CoV‐2 vaccine antigen recommendations. It should be noted that despite increasing our understanding of the epidemiology of influenza, these changes are likely to also affect the historical comparability of the data and have possibly increased the sensitivity of surveillance for other respiratory pathogens, changes that are important to be aware of for future interpretation of these different data sources (e.g., epidemic thresholds).

Our results show how NICs were able to scale up and repurpose resources (e.g., staff and equipment) following the start of the SARS‐CoV‐2 pandemic. Their ability to do so and respond to a new pathogen threat highlights the importance of strong national influenza surveillance systems globally.^37^ Currently, NICs are located in approximately 60% of WHO member states,^19^ and not all regions and populations are equally represented with only 16 NICs located in the WHO Africa region compared with 54 in the WHO Europe region.^17^ This leaves gaps in knowledge as well as an opportunity to increase global pandemic preparedness. Though our results did not show many discrepancies between the way in which surveillance in HICs was impacted by the pandemic compared with LMICs, the latter are known to have lower quality surveillance data.^22^ As such, it is important to continue efforts to strengthen these systems.

### 4.4 The future of global influenza surveillance

With SARS‐CoV‐2 being likely to continue to circulate,^38^ questions arise about the future of respiratory surveillance systems. Testing for SARS‐CoV‐2 has generally decreased, and many NICs in our survey envision a complete integration of SARS‐CoV‐2 surveillance into their existing influenza surveillance system, and WHO has outlined a transition from extensive (e.g., aimed at capturing all positive cases) to a more sustainable integration of SARS‐COV‐2 surveillance into the existing influenza sentinel surveillance system.^23^ According to a recent survey conducted by ECDC, 12 countries in Europe have already started this integration process.^39^ This is also in line with efforts to expand GISRS into GISRS^+^ by incorporating the surveillance of other respiratory viruses (e.g., respiratory syncytial virus) into the existing infrastructure.^40^ With influenza activity likely to increase again, this transition will be important to ensure that surveillance structures are back in place and functional to detect both seasonal as well as non‐seasonal influenza viruses of pandemic potential.

Based on our survey, the integration of SARS‐CoV‐2 surveillance (and other respiratory viruses) into the existing influenza infrastructure will come with several challenges. Most NICs reported that the pandemic has not negatively impacted the quality of their work. However, we also found that the NIC staff involved in surveillance had experienced a high burden of work over the past 2 years, and the transition to an integrated surveillance should aim to alleviate some of this, especially to foster the future sustainability of respiratory surveillance.^14^ Adapting existing surveillance structures would likely require the expansion of coverage of these structures (e.g., additional sentinel sites and swabbing of different patient groups) to acquire sufficient and representative samples and the implementation year‐round surveillance if this was not in place previously.^23^, ^41^ The latter is especially relevant if the seasonality of SARS‐CoV‐2 remains deviant from the typical respiratory infection season. In addition, the wider and consistent availability of multiplex PCR testing could contribute to a more efficient and sustainable integration.^23^ Though some of these adjustments have already been made according to several NICs (e.g., increased availability of multiplex PCR, NGS, increased laboratory capacity, and robustness of surveillance system), whether these changes are sustainable in the future remains to be seen. Overall, the pandemic has led to increased investments and attention to surveillance structures, and this appears to have had a positive impact on national surveillance infrastructure. However, with SARS‐CoV‐2 likely becoming endemic and the intention to integrate other respiratory viruses into national influenza surveillance systems, further investments and commitments need to be made to ensure the sustainability of these improvements.

### 4.5 Limitations

Our study provides a good understanding of the direct impact of the pandemic experienced by NICs, but it also has several limitations. The most important one is that we were only able to include 18 NICs from 14 countries in our survey. A larger and more diverse pool of NICs would be needed to make any definitive statements about the global impact the pandemic has had on national influenza systems. A second limitation is that the NICs only filled out the questionnaire at one moment in time, yet results are likely to have changed over the course of the pandemic. In order to closely monitor and collect context‐specific information, regular surveys should be carried out. Lastly, some questions, regarding increases in “robustness” or “structural” changes left room for interpretation for the respondent of the questionnaire. However, respondents indicating that they had indeed seen structural changes to the surveillance system or found their system to be more robust were invited to motivate their answer in a free text box (which all respondents made use of).

## 5 CONCLUSION

The SARS‐CoV‐2 pandemic has had a substantial impact on both the circulation of influenza and the performance of the global WHO influenza surveillance system between January 2020 and March 2022. The surveillance of influenza conducted through NICs was disrupted, initially affecting the number of samples tested for influenza. However, most NICs have shown great adaptive capacity by repurposing their activities for the monitoring of SARS‐CoV‐2 and increasing their robustness. Whether structural or not, these changes have impacted the influenza surveillance data available during the pandemic and are likely to affect the historical comparability of influenza surveillance data. The ability of NICs to rapidly respond to a global pandemic underlines the importance of capacity building and the existence of strong integrated ARI surveillance structures to monitor for new respiratory pathogens. Many NICs envision the integration of influenza, SARS‐CoV‐2, RSV, and other respiratory infections into a comprehensive respiratory infections surveillance system. The changes implemented as a result of the pandemic are likely to benefit the future development of a more integrated surveillance system. However, questions about the sustainability of the changes, especially in light of the reported increases in the burden of work, and the need for further commitments remain open.

## AUTHOR CONTRIBUTIONS

**Lisa Staadegaard:** Conceptualization; data curation; formal analysis; funding acquisition; investigation; methodology; project administration; validation; visualization; writing ‐ original draft; writing ‐ review and editing. **Marco Del Riccio:** Conceptualization; data curation; formal analysis; funding acquisition; investigation; methodology; project administration; writing ‐ review and editing. **Sytske Wiegersma:** Conceptualization; data curation; formal analysis; funding acquisition; investigation; methodology; project administration; writing ‐ review and editing. **Clotilde El Guerche‐Séblain:** Conceptualization; methodology; project administration; resources; writing ‐ review and editing. **Erica Dueger:** Conceptualization; methodology; project administration; resources; writing ‐ review and editing. **Meral Akçay:** Conceptualization; methodology; project administration; resources; writing ‐ review and editing. **Jean‐Sebastien Casalegno:** Conceptualization; data curation; formal analysis; funding acquisition; investigation; methodology; writing ‐ review and editing. **Michel Dückers:** Conceptualization; data curation; formal analysis; funding acquisition; investigation; methodology; supervision; writing ‐ review and editing. **Saverio Caini:** Conceptualization; data curation; formal analysis; funding acquisition; investigation; methodology; writing ‐ review and editing. **John Paget:** Conceptualization; data curation; formal analysis; funding acquisition; investigation; methodology; project administration; resources; supervision; validation; writing ‐ review and editing.

## CONFLICT OF INTEREST STATEMENT

JP, MDR, SC, and LS declare that Nivel has previously received RSV research grants from Sanofi Pasteur/AstraZeneca, the Foundation for Influenza Epidemiology, and the European Union's Innovative Medicines Initiative. CEG, ED, and MA are Sanofi employees and may hold shares and/or stock options in the company. The other authors have no conflicts of interest to declare.

### PEER REVIEW

The peer review history for this article is available at https://www.webofscience.com/api/gateway/wos/peer-review/10.1111/irv.13140.

## Supporting information


**Table S1.** Source (e.g. hospitalized care or general practitioner) of samples tested for influenza per NIC. *Shading indicates the proportion stemming from one source (gradient scale: dark grey = high, white = low)*
**Supplement Table 2:** Survey question: From what sources does the NIC receive samples to be tested for SARS‐CoV‐2? *Shading indicates the proportion stemming from one source (gradient scale: dark grey = high, white = low)*

## Data Availability

Data collected as part of this study is treated confidentially, and as such has not been made available. Anonymized results can be shared upon request.
