# Supplementary material for: The impact of the SARS‐CoV‐2 pandemic on global influenza surveillance: Insights from 18 National Influenza Centers based on a survey conducted between November 2021 and March 2022
Source: Influenza Other Respir Viruses. 2023 May 11;17(5):e13140. doi: 10.1111/irv.13140 (PMC10173050; doi:10.1111/irv.13140)
Supplement: Supplementary file 1 — Table S1. Source (e.g. hospitalized care or general practitioner) of samples tested for influenza per NIC. Shading indicates the proportion stemming from one source (gradient scale: dark grey = high, white = low) Supplement Table 2: Survey question: From what sources does the NIC receive samples to be tested for SARS‐CoV‐2? Shading indicates the proportion stemming from one source (gradient scale: dark grey = high, white = low) [file IRV-17-e13140-s001.docx]

**Supplement**

**Supplement Table 1**: Source (e.g. hospitalized care or general practitioner) of samples tested for influenza per NIC. *Shading indicates the proportion stemming from one source (gradient scale: dark grey = high, white = low)*

| # | Period | GP | Hospitals (%) | Home swabbing (%) | Other (%) | Explanation of ‘other’ |
| --- | --- | --- | --- | --- | --- | --- |
| 1 | Prior to pandemic | 50% | 40% | 10% | 0% |  |
| 1 | Following the pandemic | 30% | 60% | 10% | 0% |  |
| 2 | Prior to pandemic | 1-5% | 80% | 0% | 10-20% | Influenza surveillance programme - this is managed through selected GP clinics |
| 2 | Following the pandemic | 1-5% | 15-20% | 0% | 80% | This is made up of predominantly Hotel quarantine patient samples plus influenza surveillance samples |
| 3 | Prior to pandemic | 50-60% | 30-40% | 0% | 5% | Not included in surveillance. Research and projects. |
| 3 | Following the pandemic | 0% | 0% | 0% | 0% | The circuit of regional laboratories was broken – only received two samples in the 2020-2021 season. |
| 4 | Prior to pandemic | 60% | 35% | 5% | 0% | Selective studies |
| 4 | Following the pandemic | 35% | 25% | 35% | 5% | Selective studies: combination of home swabbing and GP |
| 5 | Prior to pandemic | 22% | 63% | 0% | 15% | GP not involved in sentinel surveillance |
| 5 | Following the pandemic | 9% | 90% | 0% | 1% | GP not involved in sentinel surveillance |
| 6 | Prior to pandemic | NA | NA | NA | NA |  |
| 6 | Following the pandemic | NA | NA | NA | NA |  |
| 7 | Prior to pandemic | NA | NA | NA | NA |  |
| 7 | Following the pandemic | NA | NA | NA | NA |  |
| 8 | Prior to pandemic | 3% | 3% | 0% | 94% | State/Province/Jurisdictional Public Health Labs, whom receive them from diagnostic labs or physician networks |
| 8 | Following the pandemic | 3% | 3% | 0% | 94% | Jursidiction public health labs |
| 9 | Prior to pandemic | 5% | 85% | 0% | 5% | clinical trials |
| 9 | Following the pandemic | 5% | 70% | 0% | 25% | private pathology laboratories |
| 10 | Prior to pandemic | >99% | <1% | 0% | 0% | 2008 - 2018 mix of GP sentinel surveillance and a varying subset of influenza virus positive specimens forwarded from about 15 hospital labs to the NIC; since 2018 diagnostic reports by about 20 laboratories partly overlapping with the 15 have been added to compensate for submission of limited number of specimens to the NIC; starting 2021 the NIC receives from at least the double number of laboratories influenza virus positive specimens. |
| 10 | Following the pandemic | >99% | <1% | 0% | 0% | Largely the same as before; see more details in answer to previous question. In study context we receive since late 2021 for the time being a subset of specimens from municipal health service SARS-CoV-2 testing streets that are subjected to multiplex testing including influenza virus. |
| 11 | Prior to pandemic | 0% | 50% | 0% | 50% | Diagnostic centers |
| 11 | Following the pandemic | 0% | 65% | 0% | 35% | Diagnostic centers |
| 12 | Prior to pandemic | 15% | 85% | 0% | 0% |  |
| 12 | Following the pandemic | 40% | 60% | 0% | 0% | Later in the pandemic this shifted to 10% GP and 90% Hospitals |
| 13 | Prior to pandemic | 0% | 100% | 0% | 0% | sentinel site |
| 13 | Following the pandemic | 30% | 70% | 0% | 0% |  |
| 14 | Prior to pandemic | 0% | 45% | 0% | 50% | Health Centres: clustering of ILI cases (community transmission) |
| 14 | Following the pandemic | 0% | 100% | 0% | 0% |  |
| 15 | Prior to pandemic | 20% | 60% | 0% | 20% | Primary healthcare clinics |
| 15 | Following the pandemic | 5% | 68% | 0% | 27% | Primary health care clinics |
| 16 | Prior to pandemic | 30% | 65% | 5% | 0% |  |
| 16 | Following the pandemic | 20% | 80% | 0% | 0% |  |
| 17 | Prior to pandemic | 80% | 20% | 0% | 0% |  |
| 17 | Following the pandemic | 10% | 80% | 10% | 0% |  |
| 18 | Prior to pandemic | 0% | 100% | 0% | 0% | No |
| 18 | Following the pandemic | 80% | 15-20% | 0% | 0% |  |

*GP = General practitioner, ILI = Influenza Like Illness, NIC = National Influenza Centre*

**Supplement Table 2:** Survey question: From what sources does the NIC receive samples to be tested for SARS-CoV-2? *Shading indicates the proportion stemming from one source (gradient scale: dark grey = high, white = low)*

| **#** | **GP (%)** | **Hospitals (%)** | **Home Swabbing (%)** | **Other (%)** | **‘Other' explained** |
| --- | --- | --- | --- | --- | --- |
| 1 | 20% | 60% | 0% | 20% | Institutions, Research |
| 2 | <1% | 99% | 0% | 0% |  |
| 3 | 5% | 75% | 5% | 15% | private pathology laboratories |
| 4 | 10-30% | 0-60% | 0% | 10-50% | 0-60 municipal health service testing streets for primary diagnostics; shifted to sequencing SARS-CoV-2 positive specimens; army; special studies (includes sometimes home specimen collection). |
| 5 | 0% | 100% | 0% | 0% |  |
| 6 | 5% | 68% | 0% | 27% | Public health care clinics |
| 7 | 1% | 20% | 0% | 80% | Covid collection centres |
| 8 | 70% | 10% | 20% | 0% |  |
| 9 | NA | NA | NA | NA |  |
